# Knowledge, Attitude, and Barriers to Telerehabilitation-Based Physical Therapy Practice in Saudi Arabia

**DOI:** 10.3390/healthcare8040460

**Published:** 2020-11-04

**Authors:** Saleh Aloyuni, Raed Alharbi, Faizan Kashoo, Mazen Alqahtani, Ahmad Alanazi, Msaad Alzhrani, Mehrunnisha Ahmad

**Affiliations:** 1 Department of Public Health, College of Applied Medical Sciences, Majmaah University, Al Majmaah 11952, Saudi Arabia; s.aloyuni@mu.edu.sa (S.A.); r.abdullah@mu.edu.sa (R.A.); 2 Department of Physical Therapy and Health Rehabilitation, College of Applied Medical Sciences, Majmaah University, Al Majmaah 11952, Saudi Arabia; mm.alqahtani@mu.edu.sa (M.A.); aalanazi@mu.edu.sa (A.A.); m.alzhrani@mu.edu.sa (M.A.); 3 Department of Nursing, College of Applied Medical Sciences, Majmaah University, Al Majmaah 11952, Saudi Arabia; m.ahmer@mu.edu.sa

**Keywords:** telerehabilitation, physical therapy, cross-sectional survey

## Abstract

(1) Telerehabilitation (TR) is a part of telemedicine involved in providing rehabilitation services to people in remote locations. TR in physical therapy in the kingdom of Saudi Arabia is still in its infancy and its implementation may pose different challenges in the physical therapy settings. The purpose of this nation-wide survey is to explore physiotherapists (PTs) knowledge, attitudes, and barriers towards implementation of TR in physical therapy settings; (2) Methods: A 14 item questionnaire was developed and mailed to PTs working in hospitals and rehabilitation centers across 13 provinces in Saudi Arabia; (3) Results: 347 PTs responded. Results are as follows: 58.8% (n = 204) of PTs reported that they had sufficient knowledge about TR. About31.7% (n = 110) of PTs reported that their hospital and rehabilitation center had installed TR, yet only 19.9% (n = 69) utilized the TR facility. Image-based TR was more frequently used (n = 33) as compared to sensor-based TR (n = 29) and virtual reality TR (n = 10).The main barriers were technical issues and cost related to implement TR in physical therapy settings; and (4) Conclusions: There is a relatively high number of PTs with self-reported knowledge about TR, however facilities and usage were limited. The main barriers were technical issues, staff skills, and the high cost involved in the introduction of TR in the PT-based health care settings.

## 1. Introduction

The World Health Organization has recommended running only essential rehabilitation services and suspending non-essential services to ensure safety during the COVID-19 pandemic [1]. These guidelines necessitate the suspension of most of the not-urgent physical therapy activities across the globe. Restricting clinical practice is mandatory to control the spread of infection and is a moral responsibility of every physical therapist (PT). However, such a decision for an extended period could halt the progress or may worsen the pain and disability among patients. This decision would also have a financial effect on therapists who depend on clinical practice for their livelihood. To overcome the current situation, a physical therapy regulatory body known as the World Confederation for Physical Therapy (WCPT), in association with the International Network of Physiotherapy Regulatory Authorities, suggested the implementation of telerehabilitation(TR) [2].

Telerehabilitation is a medical service provided at a distance through digital media. Such services may include assessment, diagnosis, prognosis, and treatment through the education of patients and family members. Normally telerehabilitation is provided to individuals who are living in geographically distant locations or to individuals who are not able to reach the rehabilitation center due to disability and financial constraints. As per the current scenario, the mandatory social distancing due to COVID 19 has made telerehabilitation the best method to deliver medical services and to avoid the spread of infection.

Healthcare systems across the globe have undergone a rapid transformation due to advancements in digital communication [3], consequently, changing the paper-based health record system into the electronic health records (EHR) system [4]. Initially, hospital administrative activities and employer’s data were managed through electronic means, but now even the patient management systems have adopted electronic health assessment, diagnosis, and treatment known as telemedicine [5]. TR is a form of rehabilitation using telecommunication technology to benefit patients located in remote areas [6]. TR includes health care providers such as speech pathologists, occupational therapists, biomedical engineers, physiotherapists, and other allied health care personnel. It covers all the stages of rehabilitation from assessment, diagnosis, prognosis, intervention to follow-up [7]. Rapid development in TR services stems from the desire to provide the best rehabilitation to beneficiaries irrespective of their location. Some disorders limit an individual’s mobility critically, which prevents them from attending local health services. This is often the case for people who have suffered from stroke [8], traumatic brain injury [9], developmental disorders, or progressive neurological disorders [10]. One of the most recently evolved branches of development in the field of telemedicine is telerehabilitation (TR) [11]. TR enables a disabled individual to receive health advice, assessment, and treatment from a distant expert. Traditional physical therapy involves physical touch used to guide, direct, and facilitate movement. This humane factor is lacking in TR. Advancements in the field of technology, however, have helped in some ways to overcome this limitation. The incorporation of a 3D visual reality system along with complex sensor systems, that pick up small deviations from the norm during assessment and treatment, hashelped in circumventing some of these perceptual barriers.

Kingdom of Saudi Arabia (KSA) is one of the largest countries on the African continent. The health sector has seen booming growth overthe last few decades [12]. The advanced rehabilitation services provided by several hospitals and rehabilitation centers in major cities of KSA can reach a remotely located person through TR. The only requirement for such a remotely placed health center is to have high-speed internet service and a PC-based video conferring system. In the year 2000, Experts from the Ministry of Health recommended few changes after evaluating the health care delivery system in the KSA [13]. One of the recommendationsmade by these experts was to introduce an EHR system into the hospitals [14]. At that time, only a few hospitals like King Faisal Specialist Hospital and Research Centre, and the National Guard Health Affairs Hospitals had completely implemented an EHR system [15]. For two telerehabilitation and facilities to promote this technology have been installed in the major cities in Saudi Arabia. There is no study published to date that relates to the current situation in the KSA regarding the implementation of TR-based physical therapy practice. Therefore, the purpose of this study is to explore the current knowledge, attitude, and barriers toward the implementation of TR-based physical therapy at various hospitals and centers across the KSA.

## 2. Materials and Methods

### 2.1. Study Design

The study was conducted through an online survey emailed to the PTs working in hospitals across the KSA. The approval of the study design and questionnaire was obtained from the Ministry of Health (2019-0049E). The questionnaire, along with the consent form, was sent to each of the PTs directly or to the Head of physical therapy departments of hospitals for dissemination. The contact details were obtained from the webpage of the hospital. The questionnaire was distributed to PTs working in 415 governmental, and 127 private hospitals across 13 provinces in the KSA.

### 2.2. Survey Development

Survey questions were developed by a team of experts in the department of public health, college of applied medical sciences, Majmaah University. A list of questions was framed on knowledge, attitude, and barriers intelerehabilitation. External experts reviewed the first draft of the questionnaire and provided feedback on the same. The comments were obtained from the experts and in consultation with the internal committee members, a final draft of the questionnaire was prepared and pilot-tested on 10 physical therapists (PTs) working in the hospital. Minor editing was performed to improve the grammer and readability of the questions. The final questionnaire contained a survey with 14 close-ended questions targeting three domains: General information, telerehabilitation knowledge, attitude and barriers to telerehabilitation (Appendix A).

### 2.3. Subjects

PTs working in the KSA were eligible to participate in the survey. Participation in this survey was voluntary and participants did not receive any incentives for this participation. Informed consent emailed to the participants contained all the information related to the survey and contact details of the corresponding author. Those not respomding to the survey were send reminders after every two weeks for two months. The study was approved by Ministry of Health, KSA, with Central IRB log No. 2019-0049E.

### 2.4. Statistical Analysis

Survey results were analyzed using SPSS version 20 (SPSS Inc., Chicago, IL, USA), and then descriptive statistics were obtained. The data are presented as frequency and percentage of response from the participants.

## 3. Results

**Figure 1:**
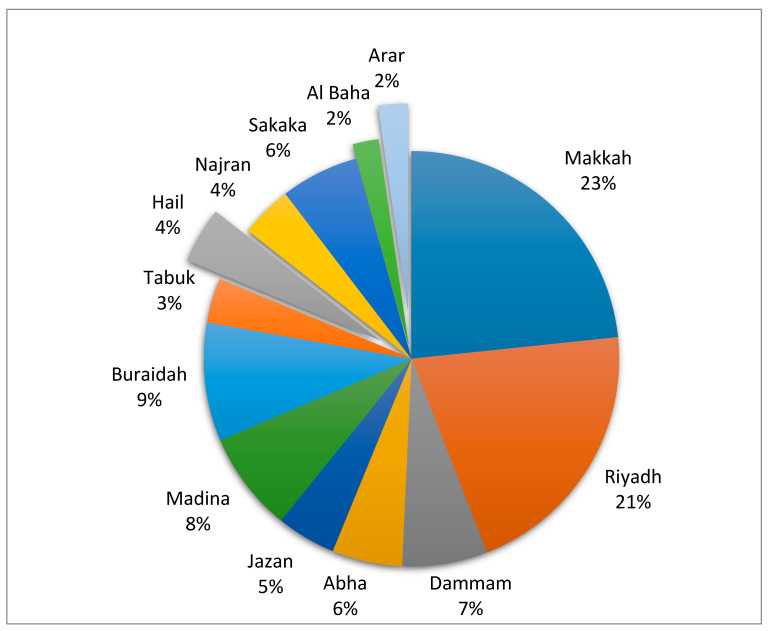
Percentage of response across the 13 provinces in Saudi Arabia.

Over 347 PTs participated in this survey (n = 347; 106 male, and 70 females) across 13 provinces in the KSA. Among 347 participants, 204 (58.8%) knew TR, and 110 (31.7%) responded that their workplace is equipped with the necessary equipment required for TR (Table 1). However, only n = 69 (19.9%) used TR at their workplace. The highest (n = 81) number of responses was from Makkah province and the lowest response was from Al Baha and Arar province of Saudi Arabia (n = 5,) (Figure 1).

**Figure 2:**
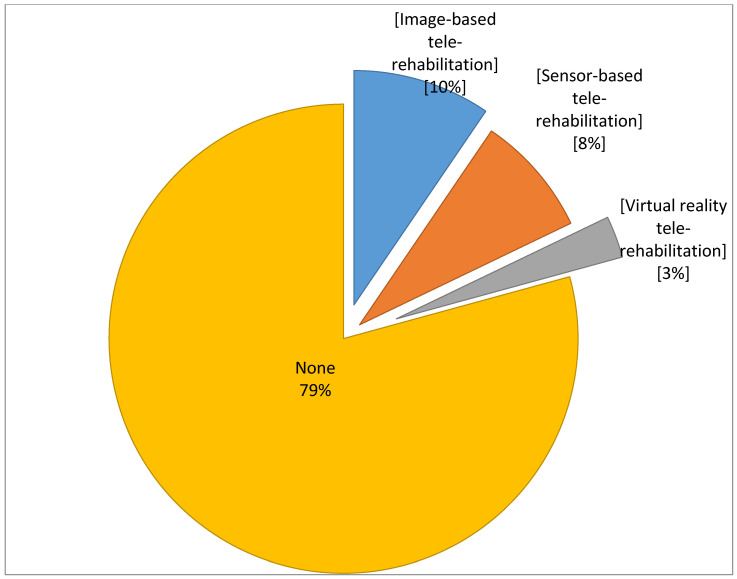
Percentage of usage of different types of telerehabilitation.

About 80.7% (n = 280) and (78.4%, n = 272) of PTs reported that the TR is reliable and valid in PT settings respectively. Furthermore, 92.2% (n = 320) agreed that the implementation of TR in the physical therapy setting will improve the quality of health care. The number of PTs using Image-based TR was highest (10% of which 33% reported from Riyadh province itself) followed by sensor-based TR (8.4%) and the least usage was Virtual reality TR (3%) (Figure 2).

**Figure 3:**
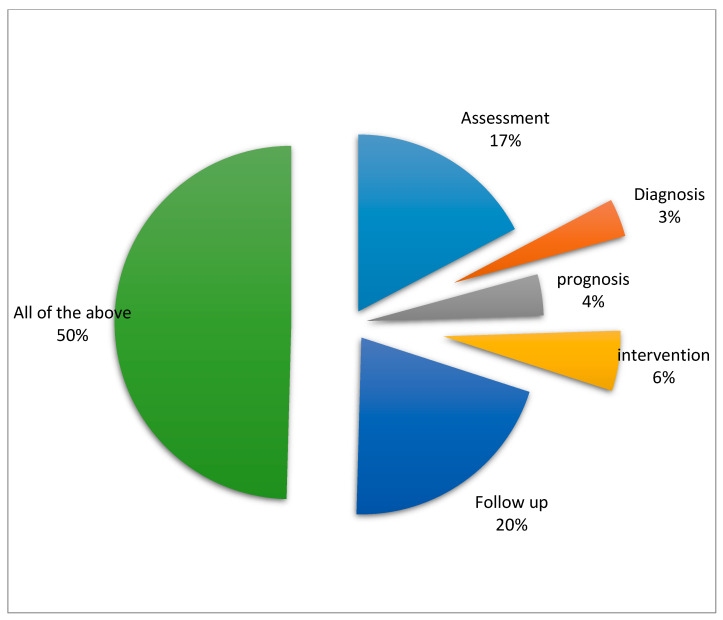
Knowledge among physiotherapists about the use of telerehabilitation across various stages of patient rehabilitation.

The PTs in the study scored highest in the general knowledge domain and more than 50% of respondents reported that the TR can be used at every stage of patient rehabilitation. The PTs reported utilizing TR in assessment (17%), Diagnosis (3%), Prognosis (4%), intervention (6%), and follow-up (20%) (Figure 3).

**Figure 4:**
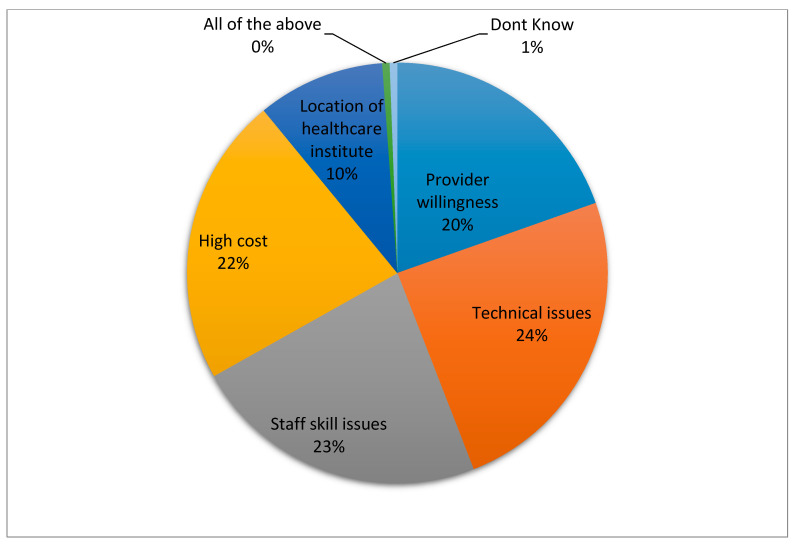
Barriers in implementing telerehabilitation in physical therapy clinical setup.

The main barriers to implementation of TR in physical therapy settings were technical issues (24%), staff skill issues (23%), and high cost (22%) provider’s willingness (20%), and location of the health care institute (10%) (Figure 4). In addition to these limitations, respondents named the attitudes of policymakers, whereas very few participants thought that the lack of skilled personnel and patient compliance factors hinder the use of telerehabilitation services.

## 4. Discussion

The study found that the majority of PTs reported having sufficient knowledge about TR. However, the usage and facilities are limited to achieve effective implementation of TR in PT settings. Participants in our research reported 9.5% usage of image-based TR. In its simplest form, the health professionals communicate through video conferencing for rehabilitation consultation [16]. Although this is a small percentage it supports the evidence from others in various countries. A rehabilitation center in Ottawa, Canada is using video-conferencing for rehabilitation consultation, specifically for patients requiring orthotic and prosthetic devices [17]. A vast number of researchers in the US had evaluated the reliability and validity of using TR for assessment and treatment of patients with neurological conditions [18] and some of the recent researchers in the University of Hong Kong have demonstrated the positive effect of video conferencing in community-based stroke rehabilitation [19]. Similarly, this study showed a high percentage of PTs reporting TR to be a reliable (80.7%) and valid (70.4%) tool in physical therapy settings. A group of researchers at the University of Queensland have developed a PC-based TR system, which enables a health professional to quantify a remotely located patient’s movement through an array of optical measurement tools connected to a high-speed internet [20]. The system was later used in a randomized controlled trial to prove that the system is capable of assessing and treating patients the same as traditional face to face rehabilitation. More than 50% of PTs in this study reported that the TR can be used for assessment, diagnosis, prognosis, treatment as well as follow-up.

Participants in this research reported 8.4% usage of sensor-based TR. Sensors such as tilt switches, gyroscope, and accelerometers are used to quantify the movement in space. Such a system evolved in the 1950s when the biomechanics laboratory at the University of California quantified patient movement [21]. Few studies are integrating the use of bio-signals in TR. Although, few pilot studies in Australia and Netherland were conducted on TR using accelerometers [22]. A researcher investigated the use of tri-axial accelerometers in elderly people to detect the risk of falls, metabolic consumption, and activity level. The device used was used to monitor real-time human movement at home [23]. The sensor-based technology seems to be a useful addition to conventional TR. There has been little published scientific research related to sensor-based TR, possibly due to the high cost of sensors.

Participants in this research reported 2.9% (n = 10) usage of virtual reality TR. Virtual reality (VR)-based TR systems use a computer-generated 3-dimensional environment, stimulating to facilitate a motor response by the patient [24]. In its simplest form, VR can be shown to the patient via a computer screen or a head-mounted VR headset worn to show a patient to perform the movement by an enriched stimulating environment [25]. The therapist involved in VR-based TR can change the virtual environment as needed for a given patient, to facilitate learning new motor skills. Limited numbers of researches are published related to VR-based TR. The research was conducted at the Massachusetts Institute of Technology using video conferencing [26] and a VR session between the patient at home and a distant therapist. The system showed positive results incertain functional activities. Similar research was conducted in New Jersey using a video conferencing system [27], a VR system coupled with a 3D sensor, and a haptic glove to provide TR consultation. Haptic gloves are used to provide resistance to the movement [28]. A home-based VR system is a futuristic rehabilitation method, as the technology becomes more advance such a system will become more practical and easy.

### Limitation

There is a limitation to our study. The nature of a web-based survey in itself carries many limitations. Respondent’s bias may be involved in self-reporting knowledge, attitude and need fornecessary equipment to implement TR in physical therapy settings. The questionnaire was introduced in English, not in the native language that might also increase the response bias based on the individual interpretation of questions in the questionnaire. Future studies must consider revising the current version of the questionnaire, particularly questions related to reliability and validity, which need to be elaborated to be dependable and acceptable respectively. However, clear instructions were provided in Arabic about the study and the questionnaire. The number of questions (n = 14) were relatively small to increase the response rate. Variables such as years of experience and qualificationswerenot asked. Data regarding the actual number of clinicians who received the e-mail could not be collected; hence, information regarding non-responders and response rate could not be determined. Further, we also suggested that the future study must include an open-ended questionnaire or interview method for respondents to explore the actual knowledge, attitude towards TR.

## 5. Conclusions

From this research, it appears that there is a relatively high number of PTsin the KSA with knowledge of TR; however, facilities and usage are limited. The main barriers reported in this study were technical issues, staff skills, and high cost involved with the introduction of TR in the PT-based health care settings. Regardless of the above-mentioned limitations, this research provides valuable evidence regarding the knowledge and understanding that PTs in the KSA have about TR and its utilization. This is especially important during the COVID-19 pandemic. Further research is suggested using a large number of therapists worldwide. This information is vital in the provision of services to our patients and the advancement of our profession in this ever-changing world.

## Figures and Tables

**Table 1:** Respondents answer to questions in the survey.

Statements	Response	Frequency (n = 347)	Percent (%)
Is the place of telerehabilitation at work well prepared?	Strongly agree	22	6.3
	Agree	39	11.2
	Neutral	139	40.1
	Disagree	33	9.5
	Strongly Disagree	114	32.9
Do you think that inclusion of telerehabilitation would improve the quality of patient care?	Strongly agree	84	24.2
	Agree	236	68.0
	Disagree	19	5.5
	Strongly Disagree	8	2.3
What do think about telerehabilitation reliability?	Strongly significant	54	15.6
	Significant	226	65.1
	Not Significant	67	19.3
Do you think that telerehabilitation is valid tool for the current health care setup?	Strongly significant	103	29.7
	Significant	169	48.7
	Not significant	36	10.4
	Strongly Not Significant	39	11.2

## Appendix A

Dear Participant, We are pleased to present this questionnaire to you, which is a research study targeting health practitioners in the field of physical therapy and rehabilitation in the Kingdom of Saudi Arabia to study **“Tele-rehabilitation services and the barriers for implementing it.”** If you are a health and rehabilitation practitioner, you are invited to participate in this questionnaire. We hope to answer all the questions accurately and objectively, because it has a significant impact on obtaining objective results supportive of this scientific research, the identity of the participant is not required and all that is in your answers will be respected and will be treated in strict confidentiality and will be used only for scientific research purposes only. If you think that the results of this search could be of interest to you, please add your email in the end of this questionnaire.It may take up to 8 minutes to complete the questionnaire. Thanking your cooperation in advance,

**Questionnaire**

**“Tele-rehabilitation services and the barriers for implementing it in Saudi Arabia.”**

**Section I: General Information.**

1. **Gender:** 1. Male. 2. Female. 2. **Specialty:** 1. Physiotherapist 2. Occupational therapist. 3. Speech pathologists. 4. Biomedical engineer. 5. Others:	3. **Place of work:**

**Section II: Knowledge of tele-rehabilitation.**

4. **What type of tele-rehabilitation you use at work?** 1. Image-based tele-rehabilitation. 2. Sensor-based tele-rehabilitation. 3. Virtual reality tele-rehabilitation. 4. Others 5. **What do you use tele-rehabilitation for?** 1. Assessment. 2. Diagnosis. 3. Prognosis. 4. Intervention 5. Follow up. 6. All of the above.	6. **Do you know tele-rehabilitation?** 1. Yes. 2. No. 7. **Do you have tele-rehabilitation at work?** 1. Yes. 2. No. 8. **Do you use tele-rehabilitation at work?** 1. Yes. 2. No. 9. **Is the place of tele-rehabilitation at work well prepared?** 1. Yes. 2. No.

**Section III: Understanding of tele-rehabilitation.**

10. **What do think about tele-rehabilitation reliability?** 1. Strongly significant. 2. Significant. 3. Not significant. 4. Strongly not significant. 11. **What do think about tele-rehabilitation validity?** 1. Strongly significant. 2. Significant. 3. Not significant. 4. Strongly not significant. 12. **Do you think that a patient can benefit of tele-rehabilitation?** 1. Strongly agree. 2. Agree. 3. Disagree. 4. Strongly disagree. 13. **Do you think that a healthcare provider can benefit of tele-rehabilitation?** 1. Strongly agree. 2. Agree. 3. Disagree. 4. Strongly disagree. 14. **What do think about the barriers of using tele-rehabilitation in Saudi Arabia?** 1. Provider willingness. 2. Technical issues. 3. Staff skill issues. 4. High cost. 5. Location of healthcare institute. 6. Others:

E.mail (Optional):
